# How many type specimens can be stored in old lesser-known herbaria with turbulent histories? – A *Juncus* case study reveals their importance in taxonomy and biodiversity research

**DOI:** 10.3897/phytokeys.153.50735

**Published:** 2020-07-16

**Authors:** Jarosław Proćków, Anna Faltyn-Parzymska, Paweł Jarzembowski, Małgorzata Proćków, Anna Jakubska-Busse

**Affiliations:** 1 Department of Plant Biology, Institute of Biology, Faculty of Biology and Animal Science, Wrocław University of Environmental and Life Sciences, ul. Kożuchowska 5b, 51-631, Wrocław, Poland; 2 Museum of Natural History, University of Wrocław, Sienkiewicza 21, 50-335 Wrocław, Poland; 3 Department of Botany, Institute of Environmental Biology, Faculty of Biological Sciences, University of Wrocław, Kanonia 6/8, 50-328, Wrocław, Poland

**Keywords:** biodiversity, conservation, historical collections, Juncaceae, *
Juncus
*, plant taxonomy

## Abstract

Many herbarium sets in Europe are still being catalogued and it is likely that many old-type collections are yet to be discovered. This research has the potential to facilitate the study of the biodiversity of many regions, especially regions for which collections are extremely scarce. This has been confirmed by a case study using *Juncus* (Juncaceae) examining the turbulent history of botanical collections at the WRSL herbarium and the evaluation of its importance to the study of taxonomy and biodiversity since 1821. The analysis revealed that the WRSL collection is rich in types (ca. 3.6%) and we identified 76 (of 78) new, historically and nomenclaturally important specimens (types, original material and so-called “topotypes”). Some of these type specimens represent duplicates of these that were stored in Berlin and destroyed during World War II. Many of the type specimens are from the United States of America, South Africa, India, and Canada. The largest number of *Juncus* type specimens stored at WRSL originate from South Africa (42.3% of all type specimens), even though *Juncus* is rare in Africa. Our study highlights that uncatalogued old collections that are under-explored and under-exploited have the potential to facilitate the discovery of specimens important for the study of biodiversity, conservation, taxonomy and nomenclature.

## Introduction

The Natural History Museum of Wrocław University (Muzeum Przyrodnicze Uniwersytetu Wrocławskiego) is the oldest natural history museum in Poland and its history dates back to 1814, when it was founded by Prof. Johann Ludwig Christian Gravenhorst as the Zoological Museum. Currently, it houses both the botanical and zoological collections. The beginning of the herbarium in its present form was the Herbarium Horti Botanici Universitatis Wratislaviensis, which was established by Prof. Ludolph Christian Treviranus in 1821 (Wiktor 2002; Wanat 2013). The Herbarium Silesiacum was independently founded by the Silesian Association of Native Culture (Schlesische Gesellschaft für Vaterländische Cultur) and, until 1945, it was housed on Tamka Island, Wrocław. It developed independently from the other botanical collections, but following the Second World War, it was merged with the main part of the herbarium.

Many distinguished botanists have worked in the WRSL herbarium (Museum of Natural History, University of Wrocław, Poland, in Polish: Zielnik WRSL), including the directors or curators of Wrocław’s botany collections, for example, Ludolf Christian Treviranus (1821–1830), Christian Gottfried Nees von Esenbeck (1830–1852), Heinrich Robert Goeppert (1852–1884), Heinrich Gustav Adolf Engler (1884–1889), Ferdinand Cohn (1884–1893) and Ferdinand Pax sen. (1893–1927). The Herbarium Silesiacum was curated by Julius Milde (1865–1870), Gustav Wilhelm Körber (1871–1885), Rudolf von Uechtritz (1886), Theodor Schube (1890–1929) and Emil Schalow (1930–1944) (Wiktor 2002).

Professor H.R. Goeppert expanded the botany collections and established the Botanical Museum (Botanisches Museum) in 1853 (Wanat 2013). The first known catalogue of the Museum (Goeppert 1884) included 26 different collections, including the Herbarium of the World, the Herbarium Silesiacum, the Herbarium Mycologicum, a wood collection and several fruit and seed sets. Goeppert also opened another museum in 1878 – The Museum of the Botanical Garden (Mularczyk 1998). In 1888, all these several botanical collections belonging to the University were moved to a building that is today located at 6/8 Kanonia Street. However, they still consisted of two separate collections (the Botanical Museum in charge of Prof. Cohn and collections of the Herbarium and the Museum of the Botanical Garden in charge of Prof. Engler). Due to the efforts of Prof. Engler, a private Silesian plant collection assembled by Rudolf von Uechtritz was purchased at this time and M. Winkler donated his herbarium to the Museum, which he had compiled for 30 years (Wiktor 2002).

At the end of nineteenth century, Ferdinand Pax (the elder) merged all the University botanical collections under the name of the Botanical Museum. His own collections were also included in the Museum at this time. Before merging, von Uechtritz’s herbarium of Silesian plants was handed over to the Herbarium Silesiacum (then still independent) on his initiative. In exchange for Uechtritz’s herbarium, the Botanical Museum later received the Herbarium Henschelianum (part of the Herbarium Silesiacum) with ca. 100,000 sheets.

As a result of these mergers and gifts, the Wrocław herbarium had acquired an extensive and significant collection of specimens from Europe (especially the Mediterranean) and the rest of the world. These were collected by botanists such as Hubert Winkler (a student of F. Pax the elder) in East Africa, Cameroon, Java, Sumatra and Borneo. In 1938, a collection of ca. 50,000 herbarium sheets (including numerous types) was donated to the Museum by Carl Adolf Georg Lauterbach, who travelled extensively in New Guinea and Melanesia. By 1914, the Herbarium had 540,000 sheets which, by 1939, had grown to ca. 600,000 sheets (Wiktor 2002; Wanat 2013). The oldest and most valuable collections of the Herbarium Silesiacum were those made by H.G. Mattuschka (1776 and 1779), A.J. Krocker (1787, 1790, 1814, 1823), A. Henschel (1830), a herbarium of fungi assembled by W.G. Schneider and an old herbarium of Paolo (Silvio) Boccone, a Cistercian monk, who moved to Wrocław in 1694 and donated his herbarium that consisted mainly of Mediterranean plants (Treviranus 1831; Rostański 1963). This herbarium is not mentioned by Stafleu et al. (1976), but is the oldest plant collection of a scientific nature in Poland. In 1935, the Herbarium Silesiacum housed over 80,000 sheets (Wanat 2013).

In autumn 1944, during the Second World War, German authorities evacuated all university botanical collections from Wrocław. The Herbarium Generale (combination of the various merged herbaria) was then located in Piotrowice castle near Kąty Wrocławskie (ca. 43 km S.W. of Wrocław), the Herbarium Lauterbachi in Siedlęcin near Jelenia Góra (ca. 95 km W. of Wrocław) and the other botanical sets in the garrison church in Oleśnica near Wrocław (ca. 27 km N.E. of Wrocław), which were unfortunately lost in a fire. The Herbarium Silesiacum was lodged, in turn, in the attic of one of the primary schools in south Wrocław (in the Tarnogaj district); however, it was not protected from destruction and the dusty and damp collection was rediscovered after the war unbound, mixed together with litter and broken glass (Wiktor 2002; Wanat 2013).

Shortly after the war, Polish authorities failed to discover traces of herbarium sets in the dilapidated buildings – these were found in the Piotrowice castle, Siedlęcin and south Wrocław only in 1946–1947, but only the Herbarium Lauterbachi was salvaged undamaged. The recovered collections were entrusted to Prof. Józef Mądalski, who was invited to Wrocław from Lviv (former Poland, now in the Ukraine). The war had damaged many of the specimens and repairs were successfully undertaken by Polish botanists. Rostański (1963) assessed the war damage in both herbaria (i.e. Herbarium Generale and Herbarium Silesiacum) as, after the war, only 200,000 herbarium sheets were discovered out of 600,000 that belonged to the University in 1939, together with 30,000 herbarium sheets from the former Herbarium Silesiacum which, in 1939, housed 80,000 sheets (it was confirmed then that the oldest Silesian flora sets of H.G. Mattuschka and A.J. Krocker had been destroyed).

Currently, the collections are estimated to contain over 515,000 sheets, including ca. 410,000 vascular plants, 27,000 bryophytes, 38,400 fungi and myxomycetes, 27,000 lichens and 12,600 algae (Mirek et al. 1997; K. Świerkosz, pers. comm., 2019). The herbarium WRSL has had a turbulent history and has enormous importance in the botanical history of Poland.

The aim of this investigation was to assess the value of the WRSL botanical collection using the genus *Juncus* as a case study. Type and other nomenclaturally and historically important specimens “hiding” in such under-appreciated collections are improtant for taxonomy, nomenclature and biodiversity studies. Using the WRSL herbarium, we address the importance of collections like WRSL as reservoirs of valuable data that are relevant to experts who are involved in taxonomic revision.

## Methods

### Assessing the significance of the WRSL collection

The WRSL herbarium is currently divided into three parts: the Herbarium Generale, the Herbarium Lauterbachi and the Herbarium Silesiacum. The Herbarium Generale (about 375,000 specimens including about 75,000 spore-bearing organisms) holds the plant and fungal material from around the world, excluding Lower Silesia, Poland, the Herbarium Lauterbachi (about 50,000 sheets) contains plants from New Guinea and Melanesia and the Herbarium Silesiacum (about 90,000 specimens) (K. Świerkosz, pers. comm., 2019) houses plants from Lower Silesia, Poland.

Generally, the importance of particular natural collections depends not only on their size, but also can be measured on the percentage or the absolute share of type specimen types (Sutory 1997). In 2017, digitalisation of the WRSL collection was initiated and was subsequently able to be accessed via GBIF.org (Świerkosz 2017); this work is on-going but only 25,000 specimens (4.9%) are currently listed in a database (K. Świerkosz, pers. comm., 2019). Therefore, we decided to assess the importance of using specimens of the genus *Juncus* (Juncaceae) stored in the Herbarium Generale (to date, no *Juncus* specimens from WRSL are included in GBIF.org database to facilitate this task). The reasons for this choice were: 1) type specimens of *Juncus* have never previously been assessed in the WRSL Herbarium; 2) the genus *Juncus* is rich in species from regions where the herbarium has geographical strengths, 311 are listed by Kirschner et al. (2002a, b) and 3) the first author of this paper is a specialist in *Juncus* taxonomy, which considerably aided the analysis of specimen status.

We evaluated the following factors (Sutory 1997): 1) the originality of the collection, including the number of types and other historically-important specimens; 2) the size of the collection, i.e. the total number of specimens; 3) the geographical scope of the collection; 4) the length of the period represented by the collection; 5) the number of duplicates and 6) the physical condition of the collection (well-prepared, well-preserved and undamaged and well-stored material with appropriate labels). Herbarium sheets with plants representing a single taxon that were gathered in the same locality and on the same date by the same collector, were regarded as duplicates. Additionally, we analysed the specimens with respect to: 1) the person who collected the material in the field; 2) the collection from which they came (i.e. to whom they belonged before accession in WRSL) and 3) the floras/exsiccatae from which they came.

We catalogued all *Juncus* specimens ourselves, paying particular attention to all types and other historical material, which we identified, based on the latest monograph (Kirschner et al. 2002a, b), from which we took the current nomenclature of the genus. The localities and dates of sets for historical collections, especially those of C.F. Ecklon & C.L.P. Zeyher and J.F. Drège, were deciphered from literature (Meyer 1832; Drège 1847, 1848; Buchenau 1875, 1890, 1906), which enabled us to recognise many *Juncus* types.

The *Juncus* sets are stored in seven herbarium boxes indexed as separate fascicles, numbered 151–157 and an extra 43 herbarium sheets were kept in a separate folder. We analysed 2,192 herbarium sheets in total. We treated a separate collection with its own label as a separate herbarium sheet, as specimens from three different localities could have been mounted on one herbarium sheet (we treated these as three separate herbarium sheets). We identified 2,222 taxonomic records, since part of the material represents mixed sets. We conducted our research from scratch, since only two *Juncus* types identified in the Herbarium Generale had been previously labelled using a red label. Thus, no other *Juncus* types stood out from other herbarium sheets. Our results were also compared with those within the Global Plants Database (plants.jstor.org, accessed on 16 Apr 2020) and additional herbaria, not mentioned by Kirschner et al. (2002a, 2002b) that store other type specimens/duplicates of names we assessed, are added to the last column of Table 1 and marked with an asterisk (*). Duplicates of selected type specimens stored at WRSL were also compared with those stored in other herbaria (present in the Global Plants Database). When comparisons were made, we considered the physical condition of specimens, quantity of materials, different annotations, kinds of labels and plant parts.

**Table 1.** A list of historically- and nomenclaturally-important *Juncus* specimens identified in the Herbarium Generale at WRSL. A sequence of species alphabetically according to the basionym *Juncus* names. No. – Successive Number; N.f. – Number of fascicle (= herbarium box) at WRSL; underline text – new findings after examination of the protologues; grey rows – indicate types that were stored in Berlin and were destroyed during the WWII; * – asterisk indicates additional herbaria where Global Plants (plants.jstor.org) records duplicates.

No.	N.f.	Kind of type and type of (basionym)	Current name	Herbarium label data (original spelling)				T: Type citation from protologue, including herbaria acronyms (according to Kirschner et al. (2002a, b)) and additional remarks (Rem.:)
				Locality (label data)	Date	*Leg.* et det.	Flora of / Herbarium	
1	151	**Authentic/original material** of *Juncus antonianus* Steud. in W. Lechler, *Berberid*. *Amer*. *Austral*. (1857: 56), nom. inval.	**Juncus balticus subsp. andicola** (Hook. 1848: 8, pl. 714) Snogerup in Snogerup, Zika & Kirschner, *Preslia* 74 (2002: 258)	PERU. S. Antonio	Jun 1854	*W. Lechler 1808* (as *Juncus andicola*, 04 Dec 1887, Fr. Buchenau)	W. Lechler, Pl. Peruvianae ed. R. F. Hochenacker / Herbarium Henschelianum	Authentic/original material: Peru, San Antonio, Jun 1954, *W. Lechler 1808*, G, GOET, K, KW*.
								Rem.: After Kirschner et al. (2002b: 74) erroneous collection date of Jun 1954 to be corrected to Jun 1854.
2	151	**Isoneotype** of *Juncus atratus* Krock., *Fl. Siles*. 1 (1787: 562)	***Juncus atratus*** Krock., *Fl. Siles*. 1 (1787: 562)	POLAND. Breslau [Wrocław], Oderdämme bei Carlowitz [Karłowice, now a settlement within Wrocław city]	10 Jul 1892	*A. Callier 721*	A. Callier Flora Silesiaca exsiccata / Herbarium Wagnerianum	T: Silesia, *A.J.Krocker*; syn: not extant; Breslau, Oderdämme bei Carlowitz [Karlowice between Wrocław and Opole, Poland], 10 Jul 1892, *A.Callier [Fl. Siles. Exs.] 721*; neo: S, designated by Kirschner et al. (2002a: 178); isoneo: L, PRC, W, WU.
								Rem.: After Kirschner et al. (2002a: 178) erroneous locality translated as ‘Karlowice [village] between Wrocław and Opole, Poland’ which is on the Stobrawa river [not Odra] and is ca. 55 km SE from the Karłowice [settlement] in Wrocław on the Odra river.
								The status of the type was corrected (iso to isoneo) in accordance with the *Shenzhen Code*.
3	154	**Syntype** of *Juncus brunneus* Buchenau, *Junc. S. Amer*. (1879: 403)	***Juncus ebracteatus*** E. Mey., *Syn. Junc.* (1822: 28)	PERU. Im paludosis prope Azangaro	Jun 1854	*W. Lechler 1749*, det. Fr. Buchenau, 22 Jan 1879	W. Lechler, Pl. Peruvianae ed. R.F. Hochenacker / Herbarium Henschelianum	T: Bolivia, La Paz, Larecaja, 2700–3800 m, *G. Mandon 1436*; syn: BM, G, K, MO, NY, P; Peru, Azangaro, *W. Lechler 1749*; syn: BR, G, GOET, K, O, P, S.
4	152	**Isotype** of *Juncus buchenaui* Sved., *Juncac. Regn. Exp.* (*Bih. Kongl. Svenska Vetensk.-Akad. Handl.*) 23(3), no 6 (1897: 9)	***Juncus marginatus*** Rostk., *De Junco* (1801: 38)	BRASILIA. Brasiliae civit. Rio Grande do Sul, Quinta	07 Dec 1892	*C.A.M. Lindman 857*	Herb. Brasil. Regnell. Musei bot. Stockholm	T: Brazil, Rio Grande do Sul, Quinta prope opp. Rio Grande, 7 Dec 1892, *C.A.M. Lindman A857*; holo: S; iso: GH, W [cf. *Juncus × buchenaui* Dörfl. 1897. an prius?].
								Rem.: After Kirschner et al. (2002a: 48) collection *No. A875* (probably to be corrected).
5	152	**Probably original material** of Juncus bufonius var. halophilus Buchenau & Fernald, *Rhodora* 6 (1904: 39)	***Juncus ranarius*** Songeon & E.P. Perrier in P.C. Billot, *Annot. Fl. France Allemagne* (1859: 192)	CANADA. Rivière du Loup	Aug 1902	*W.W. Eggleston 3036*	Plants of the Lower St. Lawrence	T: Canada, Quebec, Rivière du Loup, 2 Aug 1902, *E.F.Williams & M.L.Fernald*; holo: GH; iso: CM*, K, L, P, PH*.
								Paratypes (see protologue): Rivière du Loup, 15 Aug 1892, *G.G. Kennedy*; Rivière du Loup, 8 Aug 1902, *J.R. Churchill*, *W.W. Eggleston*, *M.L. Fernald*. See also protologue for many other paratypes.
								Rem.: After Kirschner et al. (2002b: 15) collection should be of E.F. Williams & M.L. Fernald but the herbarium label is marked as ‘Type’.
								According to the protologue the paratype should be collected by *J.R. Churchill*, *W.W. Eggleston*, *M.L. Fernald* (instead of *W.W. Eggleston* only) and with the exact collection date (8 Aug 1902).
6	152	**Holotype** of Juncus bulbosus f. submucronatus Proćków, *Ann. Bot. Fenn.* 47 (2010: 412)	**Juncus bulbosus f. submucronatus** Proćków, *Ann. Bot. Fenn.* 47 (2010: 412)	POLAND. Wrocław Leśnica, ad ripam et in aqua piscinae eutrophicae, situ meridiano-occidentali lacus	31 May 1999	*J. Proćków 990531/1*	Herbarium J. Proćków	T: Poland, Dolny Śląsk, Wrocław Leśnica, ad ripam et in aqua piscinae eutrophicae, situ meridiano-occidentali lacus, 31 May 1999, *J. Proćków*; holo: WRSL; iso: WRSL; para: B, BIL, BM, BR, C, DBN, DRAPN, E, GOET, H, HAL, HBG, KRA, L, LAU, LG, LISU, M, MA, MSB, P, PBMA, POZ, S, TRN, TUB, WA, WRSL, WSRP, ZBI.
								Rem.: After Proćków (2010: 420–423)
7–11	152	**Isotype** of Juncus bulbosus f. submucronatus Proćków, *Ann. Bot. Fenn.* 47 (2010: 412)	**Juncus bulbosus f. submucronatus** Proćków, *Ann. Bot. Fenn.* 47 (2010: 412)	POLAND. Wrocław Leśnica, ad ripam et in aqua piscinae eutrophicae, situ meridiano-occidentali lacus	31 May 1999	*J. Proćków 990531/2 to 6*	Herbarium J. Proćków	T: Poland, Dolny Śląsk, Wrocław Leśnica, ad ripam et in aqua piscinae eutrophicae, situ meridiano-occidentali lacus, 31 May 1999, *J. Proćków*; holo: WRSL; iso: WRSL; para: B, BIL, BM, BR, C, DBN, DRAPN, E, GOET, H, HAL, HBG, KRA, L, LAU, LG, LISU, M, MA, MSB, P, PBMA, POZ, S, TRN, TUB, WA, WRSL, WSRP, ZBI.
								Rem.: After Proćków (2010: 420–423).
12	152	**Paratype** of Juncus bulbosus f. submucronatus Proćków, *Ann. Bot. Fenn.* 47 (2010: 412)	**Juncus bulbosus f. submucronatus** Proćków, *Ann. Bot. Fenn.* 47 (2010: 412)	GERMANY. Leipzig, Dahlen, 2. Teich in Richtung Schmannewitz, Teichschlamm.	03 Aug 1984	*Peter Gutte 34378* (WRSL 69420)	Flora des Bezirkes Leipzig.	T: Poland, Dolny Śląsk, Wrocław Leśnica, ad ripam et in aqua piscinae eutrophicae, situ meridiano-occidentali lacus, 31 May 1999, *J. Proćków*; holo: WRSL; iso: WRSL; para: B, BIL, BM, BR, C, DBN, DRAPN, E, GOET, H, HAL, HBG, KRA, L, LAU, LG, LISU, M, MA, MSB, P, PBMA, POZ, S, TRN, TUB, WA, WRSL, WSRP, ZBI.
							Herb. Univ. Lipsiensis. Pflanzen der DDR	
								Rem.: After Proćków (2010: 420–423).
13	152	**Paratype** of Juncus bulbosus f. submucronatus Proćków, *Ann. Bot. Fenn.* 47 (2010: 412)	**Juncus bulbosus f. submucronatus** Proćków, *Ann. Bot. Fenn.* 47 (2010: 412)	CZECH REPUBLIC. Bohemia meridionalis, distr. České Budějovice; ad margines turfosas stagnorum prope rivulum Borovnický potok haud procul ab vico Borovnice, copiose, ca. 450 m s. m.	24 Aug 1962	*J. Kučera 154* (WRSL 26580)	Plantae Čechoslovacae Exsiccatae. Cura Sectionis Botanicae Musei Nationalis Pragae Editae. Centuria II	T: Poland, Dolny Śląsk, Wrocław Leśnica, ad ripam et in aqua piscinae eutrophicae, situ meridiano-occidentali lacus, 31 May 1999, *J. Proćków*; holo: WRSL; iso: WRSL; para: B, BIL, BM, BR, C, DBN, DRAPN, E, GOET, H, HAL, HBG, KRA, L, LAU, LG, LISU, M, MA, MSB, P, PBMA, POZ, S, TRN, TUB, WA, WRSL, WSRP, ZBI.
								Rem.: After Proćków (2010: 420–423).
14	152	**Paratype** of Juncus bulbosus f. submucronatus Proćków, *Ann. Bot. Fenn.* 47 (2010: 412)	**Juncus bulbosus f. submucronatus** Proćków, *Ann. Bot. Fenn.* 47 (2010: 412)	POLAND. distr. Siedlce, Krzymosze, na obnażonej ziemi w borze bagiennym obok toru [on bare soil in marshy forest next to a railway track].	27 Jul 1974	*Z. Głowacki s. n.* (WRSL 35948)	Zielnik Zakładu Biologii Wyższej Szkoły Nauczycielskiej w Siedlcach	T: Poland, Dolny Śląsk, Wrocław Leśnica, ad ripam et in aqua piscinae eutrophicae, situ meridiano-occidentali lacus, 31 May 1999, *J. Proćków*; holo: WRSL; iso: WRSL; para: B, BIL, BM, BR, C, DBN, DRAPN, E, GOET, H, HAL, HBG, KRA, L, LAU, LG, LISU, M, MA, MSB, P, PBMA, POZ, S, TRN, TUB, WA, WRSL, WSRP, ZBI.
								Rem.: After Proćków (2010: 420–423).
15	152	**Isolectotype** of *Juncus caespiticius* E. Mey. in J.G.C. Lehmann, *Pl. Preiss.* 2 (1846: 47)	***Juncus caespiticius*** E. Mey. in J.G.C. Lehmann, *Pl. Preiss.* 2 (1846: 47)	AUSTRALIA. ad fluvium Canning, Perth, novae Hollandiae.	02 Nov 1839	*Preis.* (*L. Preiss*) *1733*	Herbarium Schumann	T: [Western Australia, Perth, Canning R.] ad fluvium Canning (Perth) novae Hollandiae, 2 Nov 1839, *L.Preiss [Pl. Austral. Occid.] 1733*; lecto: W, designated by Kirschner et al. (2002a: 38); isolecto: BM, BREM, G*, K, L, LD*, MEL, NSW, P, W.
								Rem.: The status of the type corrected (iso to isolecto) in accordance with the *Shenzhen Code.*
16	155	**Isotype** of *Juncus caffer* Bertol., *Mem. Reale Accad. Sci. Ist. Bologna* 3 (1851: 253, Pl. 19, fig. 3).	***Juncus kraussii*** Hochst. in C. Krauss *Flora* 28 (1845: 342) **subsp. kraussii**	MOZAMBIQUE. ‘Inhambane Mozambici’	06 Dec 1848	*Fornasinio s.n.*		T: Mozambique, ’Inhambane Mocambici’, 6 Dec 1848, *Fornasinio*, holo: BOLO.
17	152	**Syntype** of Juncus capensis subsp. angustifolius var. ecklonii Buchenau, *Monogr. Junc.*	***Juncus capensis*** Thunb., *Prodr. Pl. Cap.* 1 (1794: 66)	SOUTH AFRICA. Paludosa ad pedem montis diaboli	19 & 28 Nov 1827 [after Buchenau 1875: 485]	*C.F. Ecklon 35* (as Juncus capensis Thbg. subsp. angustifolius var. eckloni Buchn, det. Fr. Buchenau, 11 Jan 1875)	Herbarium Henschelianum	T: Cape, Teufelsberg, *C.F. Ecklon 897*, *Unio Itin.*, *no 35* [annotated by E. Meyer under no 18]; syn: BOL, JE*, S, W.
		*Cap* (1875: 485) [*Abh. Naturwiss. Ver. Bremen* 4 (1875: 485)]						Rem.: Additional remark by Buchenau (1875: 485): ‘Un. it. No. 35’.
18	152	**Syntype** of Juncus capensis subsp. angustifolius var. ecklonii Buchenau, *Monogr. Junc.*	***Juncus capensis*** Thunb., *Prodr. Pl. Cap.* 1 (1794: 66)	SOUTH AFRICA. Paludosa planitiei capensis	Dec 1827 [after Buchenau 1875: 485]	*C.F. Ecklon 899* (as Juncus capensis Thbg. subsp. angustifolius var. eckloni Buchn, det. Fr. Buchenau, 11 Jan 1875)	Herbarium Henschelianum	T: Cape, Teufelsberg, *C.F. Ecklon 897*, *Unio Itin.*, *no 35* [annotated by E. Meyer under no 18]; syn: BOL, S, W.
		*Cap* (1875: 485) [*Abh. Naturwiss. Ver. Bremen* 4 (1875: 485)]						Rem.: Kirschner et al. (2002a: 36) did not mention this type (*C.F. Ecklon 899*) but it is listed by Buchenau (1875: 485) in the protologue of the new taxon; additionally, the specimen really seen by Buchenau (with his own handwritten label); Kirschner et al. (2002a: 36) listed var. ecklonii as homotypic with Juncus capensis var. angustifolius E. Mey.; syn: JE*, W*.
19	152	**Syntype** of Juncus capensis subsp. angustifolius var. sphangnetorum f. frondescens Buchenau, *Monogr. Junc. Cap* (1875: 490) [*Abh. Naturwiss. Ver. Bremen* 4 (1875: 490)]	***Juncus capensis*** Thunb., *Prodr. Pl. Cap.* 1 (1794: 66)	SOUTH AFRICA. Cape, Tafelberg	sine dato	*J.F. Drège aa* (det. as Juncus capensis var. angustifolius E. M.), (det. as Juncus capensis Thbg. subsp. angustifolius var. sphangnetorum f. frondescens, det. Fr. Buchenau 11 Jan 1874)	Herbarium Henschelianum	T: Cape, Tafelberg, *J.F. Drège aa*; syn: K*, P, S, W; Gipfel des Tafelberges, *C.L.P. Zeyher 47*; syn: B, destroyed.
20	152	**Isolectotype** of Juncus capensis subsp. longifolius var. gracilior Buchenau, *Monogr. Junc. Cap* (1875: 483) [*Abh. Naturwiss. Ver. Bremen* 4 (1875: 483)]	***Juncus capensis*** Thunb., *Prodr. Pl. Cap.* 1 (1794: 66)	SOUTH AFRICA. Cap. B. Spei.	05 Mar 1816	*C.H. Bergius s.n.*, det. K. Sprengel (gesamm. von Bergius, det. Fr. Buchenau 11 Jan 1875)	Herbarium Henschelianum	T: Caput bonae spei, 5 Mar 1816, *Bergius*; lecto (as holo): B, destroyed, *fide* A.A. Obermeyer, *in* A.A. Obermeyer, J. Lewis & R.B. Faden, *Fl. S. Afr.* 4/2 (1985: 83); syn: W.
								Rem.: There are more specimens mentioned in the protologue of a new taxon (Buchenau, 1875: 484) thus the lectotype was designated. Isolectotype (**the only duplicate known**) rediscovered at WRSL (the specimen includes the collection date (i.e. 5 Mar 1816), as in the the protologue). The syntype (Bergius specimen at W) does not have the collection date.
								The status of the type corrected (iso to isolecto) in accordance with the *Shenzhen Code.*
21	152	**Syntype** of Juncus capensis subsp. longifolius var. gracilior Buchenau, *Monogr. Junc. Cap* (1875: 483) [*Abh. Naturwiss. Ver. Bremen* 4 (1875: 483)]	***Juncus capensis*** Thunb., *Prodr. Pl. Cap.* 1 (1794: 66)	SOUTH AFRICA. [Cape] zwischen Paarl und Franschehoek	sine dato	*J.F. Drège b* (det. as *Juncus capensis* β. *angustifolius* E. M.), (det. as J. capensis subsp. longifolius var. gracilior Buchenau, det. Fr. Buchenau 11 Jan 1875)	Herbarium Henschelianum	T: Caput bonae spei, 5 Mar 1816, *Bergius*; lecto (as holo): B, destroyed, *fide* A.A. Obermeyer, *in* A.A. Obermeyer, J. Lewis & R.B. Faden, *Fl. S. Afr.* 4/2 (1985: 83); isolecto: W.
								Rem.: A specimen not mentioned by Kirschner et al. (2002a: 37), but listed by Buchenau (1875: 484), thus it is a syntype because there are more specimens within the protologue of a new taxon; syn: S*.
								The status of the type corrected (iso to isolecto) in accordance with the *Shenzhen Code.*
22	152	**Holotype** of Juncus capensis subsp. parviflorus Buchenau, *Monogr. Junc. Cap* (1875: 491) [*Abh. Naturwiss. Ver. Bremen* 4 (1875: 491)]	***Juncus capensis*** Thunb., *Prodr. Pl. Cap.* 1 (1794: 66)	SOUTH AFRICA. ad ripas fl. Zonder Einde, Zwellendam	Nov 1836	*C. Krauss s.n.* (det. as Juncus capensis Thbg. subsp. parviflorus Buchenau, leg. Ferd. Krauss, det. Fr. Buchenau, 11 Jan 1875; det. by C. Krauss as *Juncus cephalotes* Thunb.)	Herbarium Henschelianum	T: Cape, Swellendam, Rivier Zondereinde, Nov 1838, *C. Krauss s.n.*; holo: WRSL; iso: W.
								Rem.: Buchenau (1875: 491) listed only one specimen stored at ‘Herbarium der schlesischen Gesellschaft für vaterländische Cultur und des naturhistorischen Vereines der preussischen Rheinlande und Westfalens’, i.e. in Wrocław. Thus, this holotype of the name was confirmed by the following: 1) it was observed by Buchenau on 11 Jan 1875 and 2) it is only one specimen that lacks a clearly written collection year, which was misread by Buchenau in the protologue (1875: 491) as ‘Nov 1838’, however, identical sheets (from Herbarium R. v. Uechtritz & Herbarium Schumann, both at WRSL) read ‘Nov 1836’. Compare also with A.A. Obermeyer, *in* A.A. Obermeyer, J. Lewis & R.B. Faden, *Fl. S. Afr.* 4/2 (1985: 83).
								The status of the type corrected (iso to holo (for WRSL), and lecto to iso (for W)) in accordance with the *Shenzhen Code.*
23	152	**Isotype** of Juncus capensis subsp. parviflorus Buchenau, *Monogr. Junc. Cap* (1875: 491) [*Abh. Naturwiss. Ver. Bremen* 4 (1875: 491)]	***Juncus capensis*** Thunb., *Prodr. Pl. Cap.* 1 (1794: 66)	SOUTH AFRICA. ad ripas fl. Zonder-Einde, Zwellendam	Nov 1836	*C. Krauss s.n.* (det. as *Juncus cephalotes* Thunb.)	Herbarium Schumann	Rem.: see above
24	152	**Isotype** of Juncus capensis subsp. parviflorus Buchenau, *Monogr. Junc. Cap* (1875: 491) [*Abh. Naturwiss. Ver. Bremen* 4 (1875: 491)]	***Juncus capensis*** Thunb., *Prodr. Pl. Cap.* 1 (1794: 66)	SOUTH AFRICA. ad ripas fl. Zonder-Einde, Zwellendam (Cap. B. spei.)	Nov 1836	*C. Krauss s.n.* (det. as *Juncus cephalotes* Thunb.)	Herbarium R. v. Uechtritz	Rem.: see above
25	152	**Syntype** of Juncus capitatus var. physcomitrioides Baen., *Prosp. Herb. Eur*. (1873: 4); *Schriften Königl. Phys.-Ökon. Ges. Königsberg* 14 (1873: 16).	***Juncus capitatus*** Weigel, *Observ. Bot.* (1772: 28)	POLAND. Danzig [Gdańsk], Strand bei Zoppot [Sopot]	08 Jul 1872	*C. Baenitz s.n.*	Herbarium Schumann	T: Danzig, Strand bei Zoppot [Poland, Gdańsk, Sopot], 8 Jul 1872, *K.G. Baenitz*; syn: L; additional authentic material from the same site: 5 Jul 1876, *K.G.Baenitz [Herb. Eur.] 1506* (LD, W).
26	152	Additional material from type locality) [collected by the author of the name] of Juncus capitatus var. physcomitrioides Baen., *Prosp. Herb. Eur*. (1873: 4); *Schriften Königl. Phys.-Ökon. Ges. Königsberg* 14 (1873: 16).	***Juncus capitatus*** Weigel, *Observ. Bot.* (1772: 28)	POLAND. Danzig [Gdańsk], Ad mare balticum (Zoppot [Sopot])	05 Jul 1876	*C. Baenitz 1506*	Dr. C. Baenitz, Herbarium Europaeum	T: Danzig, Strand bei Zoppot [Poland, Gdańsk, Sopot], 8 Jul 1872, *K.G. Baenitz*; syn: L; additional material from the same site, collected by the author of the name: 5 Jul 1876, *K.G.Baenitz [Herb. Eur.] 1506* (LD, W).
27	152	Additional material from type locality) [collected by the author of the name] of Juncus capitatus var. physcomitrioides Baen., *Prosp. Herb. Eur*. (1873: 4); *Schriften Königl. Phys.-Ökon. Ges. Königsberg* 14 (1873: 16).	***Juncus capitatus*** Weigel, *Observ. Bot.* (1772: 16)	POLAND. Danzig [Gdańsk], Ad mare balticum (Zoppot [Sopot])	05 Jul 1876	*C. Baenitz 1506*	Dr. C. Baenitz, Herbarium Europaeum	T: Danzig, Strand bei Zoppot [Poland, Gdańsk, Sopot], 8 Jul 1872, *K.G. Baenitz*; syn: L; additional material from the same site, collected by the author of the name: 5 Jul 1876, *K.G.Baenitz [Herb. Eur.] 1506* (LD, W).
28	156	**Syntype** of Juncus cephalotes var. minimus Hochst., *Flora* 28 (1845: 342), *p.p.*	***Juncus cephalotes*** Thunb., *Prodr. Pl. Cap.* (1794: 66)	SOUTH AFRICA. in arenos. plan. Cap.	Nov [18]38	*C. Krauss s.n.*	Herbarium R. v. Uechtritz	T: [South Africa, Cape] ‘in arenosis planitiei capensis’, Nov 1828, *C. Krauss*; syn: W, K [both mixed collections]. Rem.: The material needs to be revised because W & K contain mixed collections; after Kirschner et al. (2002a: 73), the collection date was Nov 1828 (to be corrected to Nov 1838).
29	156	**Syntype** of Juncus cephalotes var. minimus Hochst., *Flora* 28 (1845: 342), *p.p.*	***Juncus cephalotes*** Thunb., *Prodr. Pl. Cap.* (1794: 66)	SOUTH AFRICA. in arenos. plan. Cap.	Nov [18]38	*sine coll. [C. Krauss] s.n.*	Herbarium Schumann	T: [South Africa, Cape] ‘in arenosis planitiei capensis’, Nov 1828, *C. Krauss*; syn: W, K [both mixed collections].
								Rem.: Original material was from Krauss because the identical label is on a sheet from Herbarium R. v. Uechtritz where ‘Dr. Krauss’ was added; the material needs to be revised because W & K contain mixed collections; after Kirschner et al. (2002a: 73), the collection date is Nov 1828 (to be corrected to Nov 1838).
30	152	**Syntype** of Juncus cephalotes var. minimus Hochst., *Flora* 28 (1845: 342), *p.p.*	***Juncus cephalotes*** Thunb., *Prodr. Pl. Cap.* (1794: 66)	SOUTH AFRICA. in arenosis plan. Cap.	Nov [18]38	*sine coll. [C. Krauss] s.n.* (det. as Juncus cephalothes Thbg. var. varius Bchn., Fr. Buchenau, 23 Oct 1874)	Herbarium Henschelianum	T: [South Africa, Cape] ‘in arenosis planitiei capensis’, Nov 1828, *C. Krauss*; syn: W, K [both mixed collections].
								Rem.: Original material was from Krauss because the identical label is on a sheet from Herbarium R. v. Uechtritz where ‘Dr. Krauss’ was added; the material needs to be revised because W & K contain mixed collections; after Kirschner et al. (2002a: 73), the collection date is Nov 1828 (to be corrected).
31	152	**Isolectotype** of Juncus cephalotes var. ustulatus Buchenau, *Monogr. Junc. Cap* (1875: 451) [*Abh. Naturwiss. Ver. Bremen* 4 (1875: 451)]	***Juncus cephalotes*** Thunb., *Prodr. Pl. Cap.* (1794: 66)	SOUTH AFRICA. Cape, Tafelberg	Oct 1827	*C.F. Ecklon Junc. 13.*, *2.12* (as Juncus capensis var. angustifolius E. M., det. C.F. Ecklon)	Herbarium Schumann	T: South Africa, Cape, Tafelberg, Oct 1827, *C.F. Ecklon 13*; lecto: BOL, *fide* R.S. Adamson, *J. Linn. Soc.*, *Bot.* 50 (1935: 32); isolecto: W*.
32	152	**Syntype** of Juncus cephalotes var. ustulatus Buchenau, *Monogr. Junc. Cap* (1875: 451) [*Abh. Naturwiss. Ver. Bremen* 4 (1875: 451)] or/and var. varius Buchenau, *Monogr. Junc. Cap* (1875: 451) [*Abh. Naturwiss. Ver. Bremen* 4 (1875: 451)].	***Juncus cephalotes*** Thunb., *Prodr. Pl. Cap.* (1794: 66)	SOUTH AFRICA. Paludosa montis tabularis septentr.	Nov 1826	*C.F. Ecklon 901*	Herbarium Schumann	T: [South Africa, Cape] Camps Bay, *C.F. Ecklon s.n.* (BOL); syn: PRC*, S*.
								Rem.: Mixed material containing var. ustulatus Buchenau & var. varius Buchenau, mentioned in both protologues, to be analysed.
33	152	**Syntype** of Juncus cephalotes var. varius Buchenau, *Monogr. Junc. Cap* (1875: 451) [*Abh. Naturwiss. Ver. Bremen* 4 (1875: 451)].	***Juncus cephalotes*** Thunb., *Prodr. Pl. Cap.* (1794: 66)	SOUTH AFRICA. Worcester beim Waterfall	sine dato	*C.F. Ecklon & C.L.P. Zeyher Junc. 8*, *1.11* (as Juncus capensis var. minimus La Harphe, det. *Ecklon & Zeyher*)	Herbarium Schumann	T: [South Africa, Cape] Camps Bay, *C.F. Ecklon s.n.* (BOL).
								Rem.: Kirschner et al. (2002a: 73) did not mention this type, but it is listed by Buchenau (1875: 452) within the protologue of the new taxon; however Buchenau (1875: 452) indicates stunted stamens in this material.
34	153	**Syntype** of *Juncus clausonis* Trab. in J.A. Battandier & L.C. Trabut, *Fl. Algérie*, ed. 2 (1895: 84).	***Juncus striatus*** Schousb. ex E. Mey., *Syn. Junc*. (1822: 27)	ALGERIA. Ain Taya (Alger)	Jul 1889	*J.A. Battandier & L.C. Trabut 586*	Battandier et Trabut, Pl. d’Algérie	T: [Algeria] Ain Taya près Alger, Jun 1888, *L.C. Trabut*; syn: G; Jul 1889, *J.A. Battandier & L.C. Trabut 586*; syn: G, L, MPU*.
35	157	**Isotype** of *Juncus delicatulus* Steud., *Syn. Pl. Glumac.* 2 (1855: 304)	***Juncus capensis*** Thunb., *Prodr. Pl. Cap.* 1 (1794: 66)	SOUTH AFRICA. Africa australis [Cape, Grahamstown Valley]	sine dato	*J.F. Drège 1604e*	Herbarium Henschelianum	T: Africa australis [Cape, Grahamstown Valley], *J.F. Drège 1604e*; holo: P; iso: G, S, W.
36	152	**Syntype** of Juncus dregeanus var. conglomeratus Buchenau, *Monogr. Junc. Cap* (1875: 463) [*Abh. Naturwiss. Ver. Bremen* 4 (1875: 463)].	***Juncus dregeanus*** Kunth, *Enum. Pl.* 3 (1841: 344) **subsp. dregeanus**	SOUTH AFRICA. Cap. Bon. Spei (Hassagaibosch [Assegaaibos])	sine dato	*C.L.P. Zeyher* (*C.F. Ecklon & C.L.P. Zeyher*) *Junc. 10*, *26.1* (det. as Juncus cephalotes L’Harpe var. conglomerata Nees, det. Zeyher)	Herbarium Schumann	T: Hassagaibosch [Assegaaibos], *C.F. Ecklon & C.L.P. Zeyher 10*; syn: BOL, W; Albany, *C.F. Ecklon*; *syn*: *n.v.*
37	156	**Probable syntype** of *Juncus exsertus* Buchenau, *Monogr. Juncac. Cap* (1875: 435) [*Abh. Naturwiss. Vereine Bremen* 4 (1875: 435)]	***Juncus exsertus*** Buchenau, *Monogr. Juncac. Cap* (1875: 435) [*Abh. Naturwiss. Vereine Bremen* 4 (1875: 435)]	SOUTH AFRICA. Worcester, Waterfall	sine dato	*C.F. Ecklon & C.L.P. Zeyher 1. 11* (det. as *Juncus punctorius* Thbg)		T: [Cape Provinces, Swartkops River] Zwartkops Rivier, *C.L.P. Zeyher 103*; syn: B [destroyed after having been selected as type by R.S.Adamson, *J. Linn. Soc. Bot.* 50 (1935: 15)], BOL; Worcester, Waterfall, *C.F. Ecklon & C.L.P. Zeyher* [as *Juncus punctorius 1. 11] p.p.*; syn: B [destroyed], PRE; Zondagsrivier bei Graaff-Reinet [Sundays River at Graaff-Reinet], *H. Bolus 188*; syn: BOL, K*; ‘Camdeboosberg, 4–5000 Fuss’, *J.F.Drège [Juncus oxycarpus ‘c*’]; syn: W [only!].
								Rem.: *C.F. Ecklon & C.L.P. Zeyher* [as *Juncus punctorius* 1. 11] pro parte as a syntype of the name (Kirschner et al. 2002a: 239).
38	153	**Syntype** of Juncus glaucus var. acutissimus Buchenau, *Monogr. Junc. Cap* (1875: 417)	***Juncus inflexus*** L., *Sp. Pl*. (1753: 326)	SOUTH AFRICA. Cape, Wodehouse, Klein Buffels Vallei near Gaatjie	sine dato	*J.F. Drège 8796 c*	Herbarium Henschelianum	T: Cape, Wodehouse, Klein Buffels Vallei near Gaatjie, *J.F. Drège 8796c*; syn: E*, LE*, LD, S, W.
39	152	**Syntype** of Juncus inaequalis var. viridescens Buchenau, *Monogr. Junc. Cap* (1875: 455) [*Abh. Naturwiss. Ver. Bremen* 4 (1875: 455)]	***Juncus cephalotes*** Thunb., *Prodr. Pl. Cap.* (1794: 66)	SOUTH AFRICA. Worcester beim Waterfall	sine dato	*C.F. Ecklon Junc 14.*, *1.11*	Herbarium Schumann	T: South Africa, Cape, Swellendam, *C.L.P. Zeyher 4319*; syn: BOL, K*, W [*p.p.*, ut *Juncus isolepoides* Nees, *nom. inval.*]; Hottentotts-Holland, *C.L.P. Zeyher 46*; syn: BOL, W, S*; *C.F. Ecklon 14*; syn: *n.v.*
40	152	**Probable original material** of Juncus × inundatus Drejer, *Naturhist. Tidsskr*. 2 (1838: 181)	***Juncus balticus***	DENMARK. Thy, Jyllandia	sine dato	*Drejer s.n.*	ex herb. Joh. Lange / Herbarium R. v. Uechtritz	T: *n.v.* – BM*, C*, W*.
			Willd., *Ges. Naturf. Freunde Berlin Mag. Neuesten Entdeck. Gesammten Naturk*. 3 (1809: 298) **subsp. balticus × filiformis** L., *Sp. Pl*. (1753: 326)					Rem.: The protologue of Juncus × inundatus Drejer provided the following sites: Rors Klit in Thy district and at Bulbjerg (both found by Drejer) and Kollerup Klit in Vesterhanherred (found by Poulsen). However, they are cited only as geographic localities and not as specimens. Moreover, the date of collection in the protologue is July 1837. The specimen at WRSL was collected in Thy district, but no exact locality or collection date was provided; after Kirschner et al. (2002b: 141): type – *n.v.* [*non vidi*].
								After Kirschner et al. (2002b: 141) place of publication is ‘*Bot. Tidsskr*.’ to be corrected to *Naturhistorisk Tidsskrift* (*Copenhagen*), i.e. ‘*Naturhist. Tidsskr*.’.
41	154	**Isolectotype** of *Juncus involucratus* Steud. ex Buchenau, *Abh. Naturwiss. Vereine Bremen* 4 (1875: 121)	***Juncus microcephalus*** Humb., Bonpl. & Kunth., *Gen. Sp.* 1 (1816: 237 [Quarto], 190 [Folio])	PERU. Tabina	Jul 1854	*W. Lechler 2078*	W. Lechler pl. peruviana ed. R.F. Hochenacker / Herbarium Henschelianum	T: Peru, Tabina, 1854, *W.Lechler 2078*; lecto: GOET, *fide* H.Balslev, *Fl. Neotrop. Monogr.* 68 (1996: 106); isolecto: G*, K, KW*, LE*, MO, O, S. Rem.: The status of the type corrected (iso to isolecto) in accordance with the *Shenzhen Code*.
42	154	**Isolectotype** of *Juncus kotschyi* Boiss. in C.G.T. Kotschy, *Pl. Persiae Austr.* [exsiccate series edited by R.F. Hohenacker, printed label description], no. 446 (1845) & Boissier, *Diagn. Pl. Orient.*, ser. 1, 7 (1846: 101)	**Juncus fontanesii subsp. kotschyi** (Boiss.) Snogerup in K.H. Rechinger, *Fl.* *Iranica* 75 (1971: 25)	IRAN. In paludosi ad rad. M. Sabst-Buschom, pr. U. Schiras	31 May 1842	*C.G.T. Kotschy 446*	Th. Kotschy. Pl. Pers. austr. Ed. R.F. Hohenacker 1845 / Herbarium Schumann	T: [Iran] m. Sabst-Buschon pr.[ope] u.[rbem] Schiras, 31 May 1842, *C.G.T. Kotschy [Pl. Pers. Austr.] 446*; lecto: G-BOISS, *fide* S. Snogerup, *in* K.H. Rechinger, *Fl. Iranica* 75 (1971: 25); isolecto: B*, BM, CAS*, CGE, CORD*, E*, FI*, G, GOET*, HAL*, K, KW*, MO*, P, PR, S*, UPS. Rem.: The status of the type corrected (iso to isolecto) in accordance with the *Shenzhen Code*.
43	154	**Isolectotype** of *Juncus kotschyi* Boiss. in C.G.T. Kotschy, *Pl. Persiae Austr.* [exsiccate series edited by R.F. Hohenacker, printed label description], no. 446 (1845) & Boissier, *Diagn. Pl. Orient.*, ser. 1, 7 (1846: 101)	**Juncus fontanesii subsp. kotschyi** (Boiss.) Snogerup in K.H. Rechinger, *Fl.* *Iranica* 75 (1971: 25)	IRAN. In paludosi ad rad. M. Sabst-Buschom, pr. U. Schiras	31 May 1842	*C.G.T. Kotschy 446* (det. Fr. Buchenau, 31 Jan 1875, as *J. kotschyi*)	Th. Kotschy. Pl. Pers. austr. Ed. R.F. Hohenacker 1845 / Herbarium Henschelianum	T: [Iran] m. Sabst-Buschon pr.[ope] u.[rbem] Schiras, 31 May 1842, *C.G.T. Kotschy [Pl. Pers. Austr.] 446*; lecto: G-BOISS, *fide* S. Snogerup, *in* K.H. Rechinger, *Fl. Iranica>* 75 (1971: 25); isolecto: B*, BM, CAS*, CGE, CORD*, E*, FI*, G, GOET*, HAL*, K, KW*, MO*, P, PR, S*, UPS. Rem.: The status of the type corrected (iso to isolecto) in accordance with the *Shenzhen Code*.
44	154	**Isolectotype** of *Juncus kraussii* Hochst. in C. Krauss *Flora* 28 (1845: 342)	***Juncus kraussii*** Hochst. in C. Krauss *Flora* 28 (1845: 342)	SOUTH AFRICA. ad ripas Notsinakama R., distr. George	Jan 1839	*C. Krauss s.n.* (*C. Kraussii* Specimen authenticum, Fr. Buchenau, 11 Jan 1875)	Herbarium Henschelianum	T: South Africa, George Distr., Notsinakama R., Jan 1839, *C.Krauss*; lecto: G-BOIS, *fide* S.Snogerup, *Willdenowia* 23 (1993: 57); isolecto: M, TUB*. Rem.: The status of the type corrected (iso to isolecto) in accordance with the *Shenzhen Code*.
45	154	**Isolectotype** of *Juncus kraussii* Hochst. in C. Krauss *Flora* 28 (1845: 342)	***Juncus kraussii*** Hochst. in C. Krauss *Flora* 28 (1845: 342)	SOUTH AFRICA. ad ripas Notsinakama R., distr. George	Jan 1839	*C. Krauss s.n.*	Herbarium Schumann	T: South Africa, George Distr., Notsinakama R., Jan 1839, *C.Krauss*; lecto: G-BOIS, *fide* S.Snogerup, *Willdenowia* 23 (1993: 57); isolecto: M, TUB*. Rem.: The status of the type corrected (iso to isolecto) in accordance with the *Shenzhen Code*.
46	154	**Isotype** of *Juncus lomatophyllus* Spreng., *Neue Entdeck. Pflanzenk*. 2 (1821: 108)	***Juncus lomatophyllus*** Spreng., *Neue Entdeck. Pflanzenk*. 2 (1821: 108)	SOUTH AFRICA. Cap. B. Spe.	sine dato	*C.H. Bergius s.n.* (*J. lomatophyllus* Spreng., Bergius’sches Exemplar, bestimmt von K. Sprengel, 11 Jan 1875, det. Fr. Buchenau)	Herbarium Henschelianum	T: ‘in promontorio bonae spei’ [Cape Peninsula], *Bergius*; holo: B, destroyed.
								Rem.: After Kirschner et al. (2002a: 31): holotype – B, destroyed. Isotype (**the only duplicate known**) rediscovered at WRSL.
47	156	**Syntype** of *Juncus mauritanicus* Trab., *Bull. Soc. Bot. France* 34 (1887: 396)	***Juncus punctorius*** L. f., *Suppl. Pl.* (1781: 208)	ALGERIA. Aïn el Hadjar [Oran]	20 Jul 1887	*J.A. Battandier & L.C. Trabut 294*	Battandier et Trabut, Pl. d’Algérie	T: [Algeria, Oran] Aïn el Hadjar, 1100 m, 20 Jul 1887; *J.A. Battandier & L.C. Trabut [Pl. Alger.] 294*; syn: G, L, MPU*, PR, WU; [Algeria] Batna, *B.Balansa [Pl. Algér.] 739*; syn: *n.v.*
48	156	**Authentic/original material** of *Juncus minae* Strobl ex Nyman, *Consp. Fl. Eur*. (1882: 749), *nom. inval*.	***Juncus pygmaeus*** Rich. ex Thuill., *Fl. Env. Paris*, ed. 2 (1800: 178)	ITALY. Ad oram maris Tyrrheni prope Finale	11 Apr 1874	*P. Gabriel Strobl s.n*	Flora nebrodensis / Herbarium M. Winkler	Authentic/original material: [Italy, Sicily] Flora Nebrodensis, prope Finale, *G. Strobl* (K, PR)
49	156	**Authentic/original material** of *Juncus minae* Strobl ex Nyman, *Consp. Fl. Eur*. (1882: 749), *nom. inval*.	***Juncus pygmaeus*** Rich. ex Thuill., *Fl. Env. Paris*, ed. 2 (1800: 178)	ITALY. Ad oram maris Tyrrheni prope Finale	11 Apr 1874	*P. Gabriel Strobl s.n.* (det. Uechtritz, as *J. pygmaeus* Th.)	Flora nebrodensis / Herbarium R. v. Uechtritz	Authentic/original material: [Italy, Sicily] Flora Nebrodensis, prope Finale, *G. Strobl* (K, PR)
50	155	**Isolectotype** of *Juncus monticola* Steud., *Syn. Pl. Glumac.* 2 (1855: 301)	***Juncus wallichianus*** J. Gay ex Laharpe, *Essai Monogr. Jonc.* (1825: 51)	INDIA. In montibus Nilagiri	sine dato	*R.F. Hohenacker 951*	Pl. Indiae or. (M. Nilagiri) Ed. R.F. Hohenacker. 1851 / Herbarium Henschelianum	T: [India] in montibus Nilagiri, *R.F. Hohenacker [Pl. Ind. Orient.] 951*; lecto: P, *fide* K.L. Wilson & L.A.S. Johnson, *Telopea* 9 (2001: 364); isolecto: E, G*, JE*, K, L, MPU*, P, PR, S*, W. Rem.: The status of the type corrected (iso to isolecto) in accordance with the *Shenzhen Code*.
51	155	**Isolectotype** of *Juncus monticola* Steud., *Syn. Pl. Glumac.* 2 (1855: 301)	***Juncus wallichianus*** J. Gay ex Laharpe, *Essai Monogr. Jonc.* (1825: 51)	INDIA. In montibus Nilagiri	sine dato	*R.F. Hohenacker 951*	Pl. Indiae or. (M. Nilagiri) Ed. R.F. Hohenacker. 1851 / Herbarium Felsmann	T: [India] in montibus Nilagiri, *R.F. Hohenacker [Pl. Ind. Orient.] 951*; lecto: P, *fide* K.L. Wilson & L.A.S. Johnson, *Telopea* 9 (2001: 364); isolecto: E, G*, JE*, K, L, MPU*, P, PR, S*, W. Rem.: The status of the type corrected (iso to isolecto) in accordance with the *Shenzhen Code*.
52	155	**Isolectotype** of *Juncus monticola* Steud., *Syn. Pl. Glumac.* 2 (1855: 301)	***Juncus wallichianus*** J. Gay ex Laharpe, *Essai Monogr. Jonc.* (1825: 51)	INDIA. In montibus Nilagiri	sine dato	*R.F. Hohenacker 951*	Pl. Indiae or. (M. Nilagiri) Ed. R.F. Hohenacker. 1851 / Herbarium R. v. Uechtritz	T: [India] in montibus Nilagiri, *R.F. Hohenacker [Pl. Ind. Orient.] 951*; lecto: P, *fide* K.L. Wilson & L.A.S. Johnson, *Telopea* 9 (2001: 364); isolecto: E, G*, JE*, K, L, MPU*, P, PR, S*, W. Rem.: The status of the type corrected (iso to isolecto) in accordance with the *Shenzhen Code*.
53	151	**Syntype** of *Juncus multibracteatus* Tineo in G. Gussone, *Fl. Sicul. Prodr. Suppl.* (1832: 105)	***acutus*** L., *Sp. Pl.* (1753: 325) **subsp. acutus**	ITALY. In humentibus Castronuovo	sine dato	*Todaro 556*	Todaro Flora Sicula exiccata / Herbarium M. Winkler	T: [Italy] ‘In humentibus Castronuovo’, *A. Todaro 556*; syn: BM, BR*, FI, K, W.
54	153	**Probable original material** of Juncus × obotritorum Rothm., *Wiss. Zeitschr. Univ. Greifswald* 14 (1965: 79)	**Juncus × obotritorum** Rothm. *Wiss. Zeitschr. Univ. Greifswald* 14 (1965: 79) (= J. balticus Willd. subsp. balticus × J. effusus L. subsp. effusus)	GERMANY. Prov. Mecklenburg, Dünenmoor zwischen Wustrow und Dierhagen/Fischland-Darss	15 Sep 1961	*U. Schneider s.n.*	Flora Germanica / Herbarium Ulrike Schneider	T: [Germany, Mecklenburg] inter Wustrow et Dierhagen prope Ribnitz Megalopolitanae, 16 Sep 1961, *W. Rothmaler & U. Schneider*; holo:
								*n.v.* [not given in the protologue; probably JE or GFW]
								Rem.: After Kirschner et al. (2002b: 141) the type material was collected on 16 Sep 1961, and by *W. Rothmaler & U. Schneider*.
55	155	**Probable original material** of *Juncus obtusatus* Engelm., *Trans. Acad. Sci. St. Louis* 2 (1868: 495), *nom. illeg.*, *non* Schult. (1814), nom. illeg.	**Juncus covillei var. obtusatus** [Engelmann] C.L. Hitchc. in C.L. Hitchcock *& al*., *Vasc. Pl. Pacif. Northw.* 1 (1969: 193)	USA. California	sine dato	*H.N. Bolander s.n.*, *det. Fr. Buchenau*	Herbarium Henschelianum	T: California, Mariposa, Big Tree Grove, *H.N. Bolander [G. Engelmann*, *Herb. Junc. Bor.-Amer. Norm.] 42*;
								syn: AAU, CAS*, DAO*, G*, K*, LE*, MO, NY*, PH*, PR, USCH*; *H.N. Bolander 6028*; syn: MO.
								Rem.: A handwritten label by Fr. Buchenau.
56	155	**Syntype** of *Juncus oxycarpus* E. Mey. ex Kunth, *Enum. Pl.* 3 (1841: 336)	***Juncus oxycarpus*** E. Mey. ex Kunth, *Enum. Pl.* 3 (1841: 336)	SOUTH AFRICA. Cap. b. spi. ([Cape Provinces] Liesbeek R)	sine dato	*C.H. Bergius s.n.* (det. Fr. Buchenau 11 Jan 1875 & remark by Buchenau: Bergiussches Exemplar mit der (falschen) Bestimmung v. K. Sprengel); det. by K. Sprengel as *Juncus punctorius*	Herbarium Henschelianum	T: [Cape Provinces] Liesbeek R., *C.H. Bergius*; syn: B [destroyed]; Paarl, Berg Rivier, *J.F. Drège a*; syn: K, P.
								Rem.: A syntype at WRSL is shown according to an original publication of Kunth (1841: 337). This is a new syntype (**and its only known duplicate**) discovered at WRSL.
57	156	**Syntype** of *Juncus parvulus* E. Mey. ex Buchenau, *Monogr. Junc. Cap* (1875: 447) [*Abh. Naturwiss. Ver. Bremen* 4 (1875: 447)]	***Juncus cephalotes*** Thunb., *Prodr. Pl. Cap.* (1794: 66)	SOUTH AFRICA. Cape, Namaqualand, Modderfontein	05 Nov 1830	*J.F. Drège 2472b*	Herbarium Henschelianum	T: South Africa, Cape, Namaqualand, Modderfontein, 5 Nov 1830, *J.F. Drège 2472b*; syn: BM*, BOL, E*, G*, K, L, LD*, LE*, NY*, PR, S, TUB*.
58	156	**Syntype** of *Juncus persicus* Boiss., *Diagn. Pl. Orient*., ser. 1, 7 (1846: 101)	***Juncus persicus*** Boiss., *Diagn. Pl. Orient*., ser. 1, 7 (1846: 101)	IRAN. In planitie edita Kakan m. Kuh-Daëna	17 Jul 1842	*C.G.T. Kotschy 683*	Th. Kotschy. Pl. Pers. aust. Ed. R.F. Hohenacker 1845 / Herbarium Schumann	T: [Iran] Kakun M Kuh-e Dinar, *C.G.T. Kotschy 683*; syn: BM, CGE, E, FI*, G, KW*, LE*, MO*, PR, WAG*.
59	156	**Syntype** of *Juncus persicus* Boiss., *Diagn. Pl. Orient*., ser. 1, 7 (1846: 101)	***Juncus persicus*** Boiss., *Diagn. Pl. Orient*., ser. 1, 7 (1846: 101)	IRAN. In planitie edita Kakan m. Kuh-Daëna	sine dato	*C.G.T. Kotschy 683* (det. Fr. Buchenau, 04 Feb 1875)	Pers. Austr. Inl. M. / Herbarium Henschelianum	T: [Iran] Kakun M Kuh-e Dinar, *C.G.T. Kotschy 683*; syn: BM, CGE, E, FI*, G, KW*, LE*, MO*, PR, WAG*.
60	156	**Syntype** of *Juncus pictus* Steud., *Syn. Pl. Glumac.* 2 (1855: 305)	***Juncus pictus*** Steud., *Syn. Pl. Glumac.* 2 (1855: 305)	SOUTH AFRICA. Cape, Namaqualand, Kamiesberg, Leliefontein	sine dato	*J.F. Drège 2472a*	Herbarium Henschelianum	T: South Africa, Cape, Namaqualand, Kamiesberg, Leliefontein, *J.F. Drège 2472a*; syn: BM*, BOL, E*, G, K, KW*, L, LD*, NY*, P, PR, S.
61	156	**Syntype** of Juncus sikkimensis var. pseudocastaneus Lingelsh., *in* W.Limpricht, *Repert. Spec. Nov. Regni Veg. Beih.* 12: 316 (1922)	***Juncus sikkimensis*** Hook. f., *Fl. Brit. India* 6 (1892: 399)	CHINA/INDIA [?]. Tatsienlu [Kangding]-Dawo [Dawu]. Gata (Tailing) auf der Passalm Dshaschi la ka [Tschaschilaka] (Hai tse schan) am Dshará (Iara ri), 4360 m	02 Jul 1914	*W. Limpricht 1869*, det. Lingelsheim, as Juncus sikkimensis var. pseudocastaneus Lingelsh. (on the additional label)	Flora von Ost-Tibet	T: Ngata (Taining), Tschaschilaka, zwischen Tatsienlu [Kangding] und Dawo [Dawu], Hai tse schan am Dshara, 2 Jul 1914, *W. Limpricht 1869*; syn: WRSL, *n.v.*, WU.
								Rem.: The specimen at WRSL is mentioned by Kirschner et al. (2002a: 126) but marked as *n.v.* [*non vidi*].
62	152	**Isolectotype** of *Juncus ranarius* Songeon & E.P. Perrier *in* P.C. Billot, *Annot. Fl. France Allemagne* (1859: 192)	***Juncus ranarius*** Songeon & E.P. Perrier *in* P.C. Billot, *Annot. Fl. France Allemagne* (1859: 192)	FRANCE. Moutiers (Savoie)	31 Jun & 24 Aug 1858	*Perrier 1787* (det. J. Stasiak, 29 Jan 1975, as *Juncus ambiguus* Guss. = *J. ranarius* Song. et Perr.)	Reliquiae Mailleanae / Herbarium M. Winkler	T: France, Savoie, Moutiers, 31 Jun & 21 Aug 1858, *A. Perrier*; lecto: P, *fide* T.A. Cope & C.A. Stace, *Watsonia* 12 (1978: 123); isolecto: BM*, G, K, LD, W.
								Rem.: The status of this isolectotype should be validated while taking into account the following: 1) the analysis of the lectotype at P and 2) whether the lectotypification by Cope & Stace (1978: 123) is valid (the researchers did not specify which specimen at P they selected as a type and the original material of the name is usually very extensive).
								Kirschner et al. (2002b: 15) erroneously noted the page of the lectotype indication as 127 and it should be corrected to 123.
63	156	**Syntype** of Juncus rupestris f. robusta Buchenau, *Monogr. Junc. Cap* (1875: 442) [*Abh. Naturwiss. Ver. Bremen* 4 (1875: 442)]	***Juncus rupestris*** Kunth, *Enum. Pl.* 3 (1841: 344)	SOUTH AFRICA. Cape, Kamiesberge, Eselsfontein	sine dato	*J.F. Drège 2471a*	Herbarium Henschelianum	T: South Africa, Cape, Kamiesberge, Eselsfontein, *J.F. Drège 2471a*; syn: BOL, E*, G, K, LD, PR, S.
64	156	**Isolectotype** of *Juncus schimperi* Hochst. ex A. Rich., *Tent. Fl. Abyssin.* 2 (1851: 338)	***Juncus punctorius*** L. f., *Suppl. Pl.* (1781: 208)	ETHIOPIA. In ripis uliginosis Adoam	01 Dec 1837	*W. Schimper 56* (det. Fr. Buchenau, 11 Jan 1875 as *Juncus punctorius* Thbg.)	Schimperi iter Abyssinicum, Sectio prima: plantae Adoënses / Herbarium Henschelianum	T: [Ethiopia]. In ripis uliginosis prope Adoam [Adua], 1 Dec 1837, *W. Schimper [C.F. Hochstetter*, *Herb. Un. It. Abyss.] 56*; lecto: P [as ’holo’], *fide* K.A. Lye, *in* S. Edwards, Sebsebe D. & I. Hedberg, *Fl. Ethiop. & Eritr*. 6 (1997: 389); isolecto: BR*, G*, HAL*, M*, MPU*, K, KW*, LG*, S*, TUB*, WAG*, WU. Rem.: The status of the type corrected (iso to isolecto) in accordance with the *Shenzhen Code*.
65	154	**Syntype** of *Juncus schlagintweitii* Buchenau, *Nachr. Königl. Ges. Wiss. Göttingen Geschäftl. Mitt.* 13 (1869: 255)	***Juncus himalensis*** Klotzsch *in* J.F. Klotzsch & C.A.F. Garcke, *Bot. Ergebn. Reise*	INDIA. Western Himálaya, prov. Gărhvál, Nélong viâ Múkba across the Damdár or Hat ka Tsáũra Pass tu Ussilla in the Tons Valley	26 Sep to 06 Oct 1855	*A. & H. Schlagintweit 9708*, det. Fr. Buchenau	Herbarium Schlagintweit from India and High Asia	T: [Kashmir] Tibet, Dras, ‘Matai up to the Tsoje Pass’, 14 Oct 1868, *A. & H. Schlagintweit 6668*; syn: W, US*; India, Garhwal, ‘Nelong via Mukba across the Damdar’, 6 Oct 1855; *A. & H.Schlagintweit 9708*; syn: *n.v*.
			*Waldemar* (1862: 60, tab. 97)					
66	156	**Syntype** of *Juncus schlechteri* Buchenau, *Bot. Jahrb. Syst.* 24 (1898: 459)	***Juncus cephalotes*** Thunb., *Prodr. Pl. Cap.* (1794: 66)	SOUTH AFRICA. Terra Capensis, Regio occidentalis, Bain’s Kloof	Nov 1896	*F.R. Schlechter 9154*	PlantaeSchlechterianae Austro-Africanae	T: South Africa, Cape, Bain’s Kloof, *F.R. Schlechter 9154*; syn: BM*, BOL, BR*, E*, G*, L, LD, LE*, PR, PRE, S, WAG*.
67	157	**Isotype** of *Juncus singularis* Steud., *Syn. Pl. Glumac.* 2 (1855: 302)	***Juncus singularis*** Steud., *Syn. Pl. Glumac.* 2 (1855: 302)	SOUTH AFRICA. Cape, between Vanstadensberg and Bethelsdorp	1830	*J.F. Drège 1604b*	Herbarium Henschelianum	T: Cape, between Vanstadensberg and Bethelsdorp 1830, *J.F. Drège 1604b p.p*. [some gatherings with *Juncus dregeanus*]; holo: P; iso: B [destroyed, but picture deposited at W], G, S, W.
								Rem.: Mentioned by Kirschner *et al.* (2002a: 57) as a doubtful taxon.
68	156	**Syntype** of *Juncus sonderianus* Buchenau, *Monogr. Junc. Cap* (1875: 476) [*Abh. Naturwiss. Ver. Bremen* 4 (1875: 476)]	***Juncus sonderianus*** Buchenau, *Monogr. Junc. Cap* (1875: 476) [*Abh. Naturwiss.*	SOUTH AFRICA. [Cape] Port Elizabeth	sine dato	*J.F. Drège e* (det. F. Buchenau as *Juncus sonderianus* Buchenau, 11 Jan 1875; det. J.F. Drège as *cap.* β. *angustifol.* E.M.)	Herbarium Henschelianum	T: [Cape] Port Elizabeth, *J.F. Drège e*; syn: E*, G, HBG*, K, LD, LE*, P, S*, W [’J.F.Drège e’ was generally proposed as a type by Adamson, *J. Linn. Soc.*, *Bot.* 50 (1935: 26)]; [Cape] bei Cap Recief und Port Elizabeth, *C.F. Ecklon & C.L.P. Zeyher 9*; syn: BOL, LD*, W, S; *C.F. Ecklon & C.L.P. Zeyher 780*; syn: *n.v.* – W*.
			*Ver. Bremen* 4 (1875: 476)]					
69	156	**Isolectotype** of *Juncus sparganiifolius* Boiss. & Kotschy ex Buchenau, *Krit. Verz. Juncac.* (1879: 88)	***Juncus sparganiifolius*** Boiss. & Kotschy ex Buchenau, *Krit. Verz. Juncac.* (1879: 88)	TURKEY. In alvei glareosis dispersa et rara supra Ursusa pagum (Hatay, Arsuz)	02 Jul 1862	*C.G.T. Kotschy 102*	Th. Kotschy, Pl. Syriae bor. ex Amano occidentali supra Arsus 1862	T: Plantae Syriae borealis ex Amano occidentali supra Arsus, supra Ursusa pagum [Turkey, Hatay, Arsuz], 2 Jun 1862, *C.G.T. Kotschy 102*; lecto: Z, *fide* S. Snogerup, *in* P.H. Davis, *Fl. Turkey* 9 (1986: 19); isolecto: BM, G*, JE*, K, L, LE*, P, W [One of four isotype specimens from W bears a note in Buchenau’s hand: ’An excellent new species’ [translated], and should be given preference]. Rem.: The status of the type corrected (iso to isolecto) in accordance with the *Shenzhen Code*.
70	156	**Isolectotype** of Juncus sprengelii Nees ex Buchenau var. gracilior Buchenau, *Monogr. Junc. Cap* (1875: 449) [*Abh. Naturwiss. Ver. Bremen* 4 (1875: 449)]	***Juncus stenopetalus*** Adamson, *J. S. African Bot.* 8 (1942: 273)	SOUTH AFRICA. Worcester, Waterfall	sine dato	*C.F. Ecklon & C.L.P. Zeyher 11*, *1.12* (det. Fr. Buchenau, as *J. sprengelii* N. ab. Es., 11 Jan 1875)	Herbarium Henschelianum	T: South Africa, Cape, Tulbagh Waterfall, *C.F. Ecklon & C.L.P. Zeyher 11*; lecto: BOL, *fide* A.A. Obermeyer, *in* A.A. Obermeyer, J. Lewis & R.B. Faden, *Fl. S. Afr.* 4/2 (1985: 88); isolecto: LD, S, W.
71	156	**Isolectotype** of Juncus sprengelii Nees ex Buchenau var. gracilior Buchenau, *Monogr. Junc. Cap* (1875: 449) [*Abh. Naturwiss. Ver. Bremen* 4 (1875: 449)]	***Juncus stenopetalus*** Adamson, *J. S. African Bot.* 8 (1942: 273)	SOUTH AFRICA. Worcester, Waterfall	sine dato	*C.F. Ecklon & C.L.P. Zeyher 11*, *1.12*		T: South Africa, Cape, Tulbagh Waterfall, *C.F. Ecklon & C.L.P. Zeyher 11*; lecto: BOL, *fide* A.A. Obermeyer, *in* A.A. Obermeyer, J. Lewis & R.B. Faden, *Fl. S. Afr.* 4/2 (1985: 88); isolecto: LD, S, W.
72	152	**Isolectotype** of *Juncus sulcatus* Hochst. *in* C. Krauss, *Flora* 28 (1845: 342)	***Juncus capensis*** Thunb., *Prodr. Pl. Cap.* 1 (1794: 66)	SOUTH AFRICA. Ad rivulos in Zitzikama, Uitenhage	Mar 1839	*C. Krauss s.n.* (det. Fr. Buchenau, as J. capensis Thbg. subsp. angustifolius var. flaccidus Bchn., f. depaup., 11 Jan 1875)	Herbarium Henschelianum	T: Cape, Uitenhage, Zitzikamma, Mar 1839, *C. Krauss s.n.*; lecto: W, *fide* Kirschner *et al.* (2002a: 36); isolecto: FI*.
73	152	**Isolectotype** of *Juncus sulcatus* Hochst. *in* C. Krauss, *Flora* 28 (1845: 342)	***Juncus capensis*** Thunb., *Prodr. Pl. Cap.* 1 (1794: 66)	SOUTH AFRICA. Ad rivulos in Zitzikama, Uitenhage	Mar 1839	*C. Krauss s.n.*	Herbarium Schumann	T: Cape, Uitenhage, Zitzikamma, Mar 1839, *C. Krauss s.n.*; lecto: W, designated by Kirschner *et al.* (2002a: 36); isolecto: FI*.
74	157	**Syntype** of Juncus sylvaticus var. multiflorus Rochel, *Pl. Banat. Rar.* (1828: 31, tab. 1) & *Juncus rochelianus* Schult. & Schult. f., *Syst. Veg*. 7(2) (1830: 1658)	***Juncus thomasii*** Ten., *App. Ind. Sem.* (1827: sine pag.)	SERBIA. Banatu [Banatus]	1815	*A. Rochel s.n.*	Herbarium R. v. Uechtritz	T: [Romania] Valle Kornia-Reva & ad pedes Kraku-Sanozy Banatus, *A. Rochel*; syn: *n.v.* – BM*, W*; Banatus, 1815, *A. Rochel*; syn: W.
75	151	**Isolectotype** of *Juncus tommasinii* Parl., *Fl. Ital.* 2 (1852: 315)	***Juncus littoralis*** C.A. Mey., *Verz. Pfl. Casp. Meer.* (1831: 34)	ITALY. […] bog, Monfalcone Grado	sine dato	*M. Tommasini s.n.*	Ex herbario Florae Illyrico-litoralis / Herbarium R. v. Uechtritz 27	T: [Italy] ‘Nei paludi presso Monfalcone, Grado’, *M.G.S. Tommasini*, lecto: FI, *fide* S. Snogerup, *Willdenowia* 23 (1993: 40).
76	157	**Isotype** of Juncus triformis var. brachystylus Engelm., *Trans. Acad. Sci. St. Louis* 2 (1868: 492)	***Juncus kelloggii*** Engelm., *Trans. Acad. Sci. St. Louis* 2 (1868: 494)	USA. Calif[ornia], Mendocino Co., Ukiah	May 1866	*H.N. Bolander & Kellogg 4646*, det. Fr. Buchenau	Herbarium Henschelianum	T: USA, California, Mendocino Co., Ukiah, May 1866, *H.N. Bolander 4646 [G. Engelmann*, *Herb. Junc. Bor.-Amer. Norm.*]; holo: MO; iso: BM*, CAS, F*, G*, GH*, K*, MIN*, NY, PH*, PR, RM*, RSA*, US, USCH*, YU*.
77	157	**Isolectotype** of Juncus triformis var. stylosus Engelm., *Trans. Acad. Sci. St. Louis* 2 (1868: 492)	***Juncus triformis*** Engelm., *Trans. Acad. Sci. St. Louis* 2 (1868: 492)	USA. Calif[ornia], Yosemite Valley, De Long’s ranch	10 Jun 1866	*H.N. Bolander & Kellogg 4864*, det. Fr. Buchenau	Herbarium Henschelianum	T: California, Yosemite Valley, De Long’s Ranch, 4000 ft. [ca. 1280 m], 10 Jun 1866, *H.N. Bolander 4864 [G.Engelmann*, *Herb. Junc. Bor.-Amer. Norm. 30*]; lecto: MO, *fide* F.J. Hermann, *Leafl. W. Bot.* 5 (1948: 114); isolecto: CAS, DAO*, G*, ISC*, K*, LE*, MICH, NEB*, NY, PH*, RM*, RSA*, US, USCH*, YU*.
78	156	**Isotype** of *Juncus valdiviae* Steud., *Syn. Pl. Glumac*. 2 (1855: 296)	***Juncus procerus*** E. Mey., *Linnaea* 3 (1828: 367)	CHILE. ad ripam fluvii Valdivia	Jan 1852	*R.A. Philippi 43* (det. Fr. Buchenau, as *Juncus procerus* E. M., 3 Dec 1878)	R.A. Philippi, Pl. chilenses, W.R.F. Hohenacker / Herbarium Henschelianum	T: Chile, Valdivia, *R.A. Philippi 43*; holo: P; iso: FI*, G, GOET, K, KW*, MO, O, P, S.

## Results

### Type and other historically-important material

**Figure 1.**
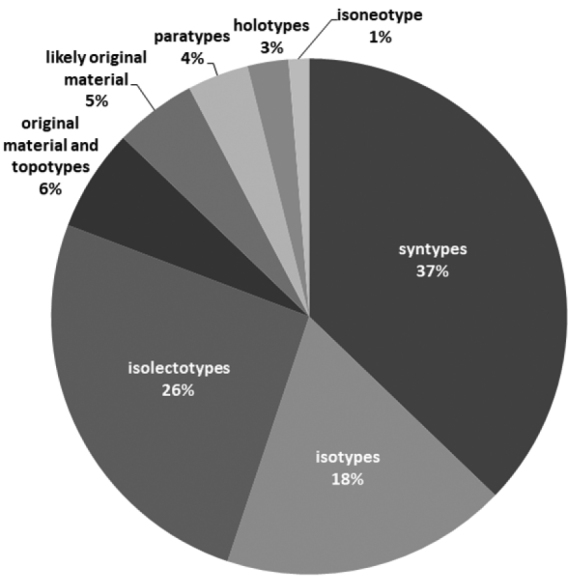
Percentage of different categories of *Juncus* specimens. Types, original material and specimens collected from the original type localities, by the author of the name (“topotypes”) at WRSL.

We found 78 specimens that are historically or nomenclaturally important (Table 1): two holotypes, 20 isolectotypes, 14 isotypes, 29 syntypes (including one probable syntype of *Juncus exsertus*Buchenau (1875: 435)), three paratypes, one isoneotype, five sheets of historically-relevant material (for names not validly published) or additional material from type localities collected by the author of the name (so-called “topotypes”) and four sheets of probable original material to be analysed in the future (Fig. 1). Holotypes, isotypes and isolectotypes constitute 46.2% of all types (and other historically- and nomenclaturally-important specimens) of *Juncus* specimens recognised at the WRSL. The most significant discovery was the identification of the three following *Juncus* types in the WRSL Herbarium (see also remarks for them in Table 1, last column of rows 46, 56, 20):

1) ISOTYPE of *Juncus lomatophyllus* Spreng. (1821: 108) [sine dato, *Bergius s.n.* (*J. lomatophyllus* Spreng., Bergius’sches Exemplar, bestimmt von K. Sprengel, 11 Jan 1875, det. Fr. Buchenau)]. – Holotype in B, destroyed. Isotype (the only duplicate known) rediscovered at WRSL.

2) SYNTYPE of *Juncus oxycarpus* E. Mey. ex Kunth (1841: 336) [sine dato, *C.H. Bergius s.n.* (det. Fr. Buchenau 11 Jan 1875 & remark by Buchenau: Bergiussches Exemplar mit der (falschen) Bestimmung v. K. Sprengel); det. by K. Sprengel as *Juncus punctorius*]. – A syntype at WRSL shown, according to the original publication of Kunth (1841: 337). This is a new syntype (and its only duplicate known) discovered at WRSL.

3) ISOLECTOTYPE of Juncus capensis var. graciliorBuchenau (1875: 483) [05 Mar 1816, *Bergius s.n.*, det. K. Sprengel (gesamm. von Bergius, det. Fr. Buchenau 11 Jan 1875), current name: *Juncus capensis* Thunb. (1794: 66)]. – Additional specimens were mentioned in the protologue of the new taxon (Buchenau, 1875: 484). Thus, the lectotype was designated (in B, destroyed). Isolectotype (the only duplicate known) was rediscovered at WRSL (the specimen includes collection date (i.e. 5 Mar 1816), which corresponds to the date included in the protologue). The syntype (Bergius specimen at W) does not include a collection date.

**Figure 2.**
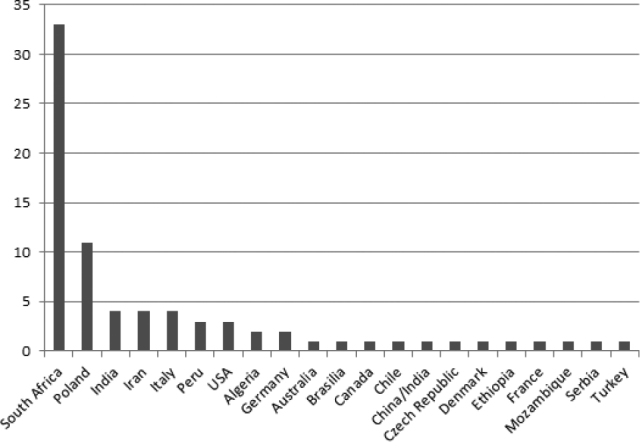
Origin of *Juncus* historically- and nomenclaturally-important specimens at WRSL according to country. Y-axis: number of herbarium sheets. Specimens most frequently originated from South Africa (42.3%). *Juncus* type specimens were collected by many distinguished botanists. Amongst these, the four individuals gathered 37.2% of *Juncus* specimens: C.F. Ecklon & C.L.P. Zeyher, C. Krauss and J.F. Drège.

The origin of *Juncus* type specimens at WRSL according to country is presented in Fig. 2.

Most of the types and other historically- and nomenclaturally-important specimens come from the following collections: Herb. Henschelianum (30 sheets, i.e. 16.2% of the *Juncus* set at WRSL – see below “A Herbarium/Collection name”), Herb. Schumann (13 sheets, 16.9%), Herb. R. v. Uechtritz (7 sheets, 1.7%), Herb. J. Proćków (6 sheets), Herb. M. Winkler (3 sheets) and others (19 sheets). Additionally, eight paratypes of J. bulbosus f. submucronatusProćków (2010: 412) are stored in the Herbarium Silesiacum at WRSL (Proćków 2010) and, thus, are not included in the statistics in this study that covers Herbarium Generale only (as a separate set of two ones at WRSL).

### Species

Approximately 70 *Juncus* species are represented in the collection, most of them from Europe. Species from the rest of the world are less numerous, but still relatively frequent: *J. capensis* Thunb., *J. subulatus* Forssk. (incl. *J. multiflorus* Desf.), *J. nodosus* L., *J. cephalotes* Thunb., *J. dichotomus* Elliott, *J. prismatocarpus* R. Br., *J. acuminatus* Michx., *J. xiphioides* E. Mey., *J. concinnus* D. Don, *J. wallichianus* J. Gay ex Laharpe (incl. *J. monticola* Steud.), *J. pelocarpus* E. Mey., *J. marginatus* Rostk., *J. microcephalus* Humb., Bonpl. & Kunth. and *J. punctorius* L.f., *J. littoralis* C.A. Mey. (as *J. tommasinii* Parl.).

### Date of collection

We found 2,193 herbarium labels with dates of collection recorded: 1,967 of these were collected before 1946, comprising ca. 89.7% of the *Juncus* set. The remaining 226 specimens were collected after 1945; 10.3% of the *Juncus* specimens.

### Collector and herbarium collection name

**Figure 3.**
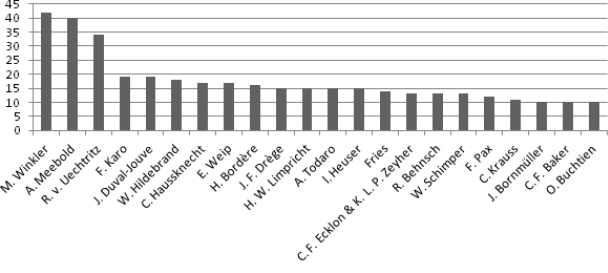
Collectors’ names. Y-axis: number of herbarium labels analysed.

In the *Juncus* set at WRSL, the sets of some individuals stand out in numbers of specimens (Fig. 3). The most outstanding collections of *Juncus* from particular included herbaria are (number of herbarium sheets are in parentheses): Herb. R. v. Uechtritz (415), Herb. M. Winkler (394), Herb. Henschelianum (185), Botanischer Tauschverein in Wien (80), Herb. Schumann (77), Herb. Wagnerianum (41), Herb. Dr. C. Baenitz (34), Herb. Emil Fiek (32), Herb. J.A. Allen (24), Reliquiae Mailleanae (24), Herb. F. Pax (21), Herbier P. Louis-Marie (20), Herb. A. Engler (18), Reliquiae Hildebrandianae (18), Herb. Felsmann (15), Herb. J. Duval-Jouve (14), Herb. Schlagintweit from India and High Asia (12), Herb. Hort. Bot. Calcuttensis (11) and Herbier Henri van Heurck (10). Almost half of the *Juncus* sp. sheets come from four individual collections. All were bought for, donated to or exchanged by the Museum. The number of duplicates in the collection is not large (4.6%, i.e. 103 out of 2,222 all taxonomic records).

### Country of collection

**Figure 4.**
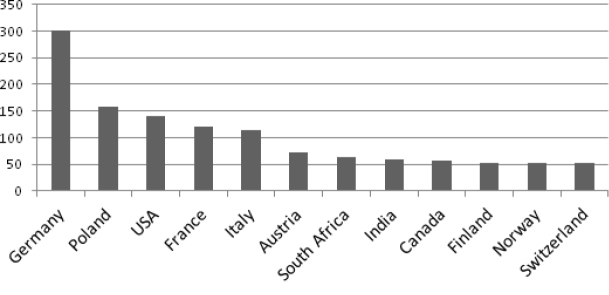
Country repsentation of *Juncus* specimens in WRSL. Y-axis: number of herbarium labels analysed. The African collection deserves particular attention (98 sheets (4.5%)), including sets from South Africa (64 sheets). The Asian collection (96 sheets) is dominated by plants from India (59). The percentage of plants from North America is as high as 10%.

Herbarium sheets from eastern Poland and Germany (defined according to their post-war borders) dominate and are shown in Fig. 4. For 336 *Juncus* sheets (15.3%), we were unable to establish the country of origin, because no or illegible information on the locality was present on herbarium labels.

### Exsiccata series

In the WRSL *Juncus* set, the following exsiccatae are particularly well-represented (the number of herbarium sheets is shown in parentheses): Rchb. Fl. germ. excurs. (incl. Rchb. Fl. Germ. n.) (37), Cyperaceae, Juncaceae, Typhaceae et Sparganiaceae Hungaricae exsiccatae (24), Reise durch das südliche Spanien 1873 (mainly of M. Winkler) (22), Flora of Sikkim (15), Pl. Indiae or[ientalis] (M. Nilagiri), ed. R.F. Hohenacker (13), Todaro Flora Sicula exiccata (11), Flora des NW. Himalaya (10).

## Discussion

The Herbarium Generale of the mid-sized WRSL herbarium is rich in specimens relevant to the nomenclature of *Juncus* and contains 78 specimens (3.6% of all *Juncus* specimens examined, see Table 1), with an average of 11 sheets per fascicle (i.e. herbarium box). Seventy-six (of 78) of these historically-important specimens (types, original material and specimens collected at the type locality by the author of the name) were not identified as such before our study. This significant number of types highlights the significance of the analysed set and of WRSL more broadly, for the study of taxonomy and nomenclature (Sutory 1997). To put this into context, the approximate percentages of types stored in other historically-important herbaria are as follows: K (5%), W (3.6%), BM (2.6%) [cited from herbaria websites, which include the total number specimens stored]. Our study revealed that the *Juncus* set at WRSL is a valuable collection globally with respect to the number of historically- and nomenclaturally-relevant specimens. Often, specimens included are associated with research conducted involving a given group of plants in the academic centre housing collections. The majority of *Juncus* specimens (ca. 89.7%) date from before the Second World War and specialists studying the genus *Juncus* did not work at WRSL during that time. This suggests that the rest of the WRSL collection might also contain similarly high percentages of such historically- and nomenclaturally-relevant specimens.

As the genus *Juncus* is rich in species (311 species, Kirschner et al. (2002a, b)), we consider that extrapolation of our results to other genera is appropriate. We assume that descriptions of taxa new to science before 1946 were equally common within most taxonomic groups and specimens belonging to different plant genera/families were sent to the WRSL herbarium equally often.

Only a small fraction of global herbarium specimens had been computerised by the end of last decade (Lughadha and Miller 2009). Despite the recent acceleration of the digitisation of herbarium collections (as of early 2015, the number of scanned specimens within the world’s largest virtual herbaria was 18.4 million), we are far from fully digitising all collections (Seregin 2016). Even a small percentage (1–2%) of computerised specimens can drastically reduce research costs and help scientists focus on collections that are likely to contain the most information-rich specimens (O’Connell et al. 2004). In herbarium management, it is cheaper to produce and distribute scans than facilitate botanist visits (Seregin 2016). Digitisation is also important because young people who do not live near a natural history museum or herbarium can access natural history data and learn to use it and this early involvement in science may cultivate a love for the study of biology (Watanabe 2019). The continued digitisation of the WRSL herbarium (currently only 4.9% digitally available) will certainly reveal new material for botanists’ use.

Our results reveal the usefulness of lesser-known herbaria not only from a national or local point of view (Lavoie 2013), but also as a source of important collections and type specimens that are not duplicated in larger facilities (Snow 2005). For *Juncus*, only two of 78 nomenclaturally-relevant specimens identified here were cited by Kirschner et al. (2002a, 2002b), so 76 of the specimens in Table 1 were unknown before this study. Holotypes, isotypes and isolectotypes constitute 46.2% of all types (and other nomenclaturally important specimens) of *Juncus* recognised at the WRSL, highlighting the nomenclature relevance of the collection. Three specimens are particularly worth highlighting here: the holotypes of *Juncus lomatophyllus* Spreng. and Juncus capensis var. gracilior Buchenau and a syntype of *J. oxycarpus* E. Mey. ex Kunth were originally stored in Berlin (the herbarium of the Botanischer Garten und Botanisches Museum Berlin-Dahlem, Freie Universität Berlin). These were destroyed during the Second World War (Hiepko 1987; Kirschner et al. 2002a) and our discovery of duplicates in WRSL will help with the correct application of these names.

Duplicates of nomenclaturally relevant specimens are often considered to be less important than holotypes, lectotypes and neotypes. Duplicates, however, may differ in physical condition, material quantity, different annotations, labelling, specimen content (plant parts, for example, young fruit vs. only a flowering twig, male vs. female flowers in diclinous plants, with roots vs. without roots) or may even represent mixed gatherings (different taxa). An isotype of *Juncus singularis* Steud. (*J.F. Drège 1604b*) at WRSL, for example, is a much larger, leafy specimen with five inflorescences, as compared with other specimens at G, P, S and W, listed and pictured at plants.jstor.org (accessed on 16 Apr 2020). Annotations by specialists can be very useful in understanding taxonomic concepts: 23 WRSL *Juncus* type specimens were annotated by Franz G.Ph. Buchenau (1831–1906), a *Juncus* specialist whose work remains unsurpassed to this day (annotations included new determinations and/or ‘specimen authenticum’ indications and were made by him throughout 1874–1875, 1878–1879 and 1887; see the ‘*Leg.* et det.’ column in Table 1). Thus, some ‘ordinary duplicates’ at WRSL are helpful for understanding taxonomists’ thinking.

We also found that many of the historically- and nomenclaturally-important *Juncus* specimens stored at WRSL originate from South Africa (42.3%). This over-representation might be explained by the origin of the collection. German botanists (together with the British and the Dutch) were a dominant force in the floristic exploration of Africa from the 17^th^ to the early 20^th^ century. The WRSL herbarium is, thus, an important resource for international researchers working on the flora of that hugely biodiverse, but still under-explored, part of the world.

## Conclusions

The history of German-Polish herbaria, including WRSL, is very turbulent. A detailed examination of *Juncus*, as a case study, confirms the value of the WRSL collection in historical terms. That a significant number of historically- and nomenclaturally-important specimens at WRSL was acquired passively (*Juncus* was of no special interest to German or Polish scientists at the time) suggests that more such specimens may be found within the collection for other genera. Digitisation and taxonomic revision of material will facilitate the confirmation of the richness of the collection.

Other large type collections contain well-preserved specimens, well-prepared catalogues (often available on-line) and are well-known to scientists. However, the WRSL collection is not only unique, as confirmed here, but not well-known to date.

Some *Juncus* type specimens, listed here, can be found easily in a large number of other collections. However, some are preserved only at WRSL because many types, previously stored in Berlin, were destroyed during the Second World War. Although we researched only a few parts of the WRSL collection, we are convinced that duplicates of many type specimens destroyed in Berlin can be found in Wrocław. Uncatalogued herbaria like WRSL with turbulent histories can be a source of collections important for the study of biodiversity.

We selected *Juncus* as a case study since the collection at WRSL covers the entire distribution range of the genus. Therefore, it likely reflects the general situation in other groups of plants in the herbarium.

Currently, many herbarium sets in Europe are still being catalogued (and many remain undigitised). However, many old collections are indeed valuable and their type and other historical collections have the potential to facilitate taxonomy and nomenclature and, in addition, enhance our knowledge of biodiversity through application of correct names.
